# A Review Over Benefits and Drawbacks of Combining Sodium Hypochlorite with Other Endodontic Materials

**DOI:** 10.2174/1874210601711010661

**Published:** 2017-12-26

**Authors:** Zahed Mohammadi, Sousan Shalavi, Amir Moeintaghavi, Hamid Jafarzadeh

**Affiliations:** 1 Iranian Center for Endodontic Research (ICER), Research Institute of Dental Sciences, Shahid Beheshti University of Medical Sciences, Tehran, Iran; 2 Private Dental Practice, Hamedan, Iran; 3 Department of Periodontics, Faculty of Dentistry, Mashhad University of Medical Sciences, Mashhad, Iran; 4 Department of Endodontics, Faculty of Dentistry, Mashhad University of Medical Sciences, Mashhad, Iran

**Keywords:** Sodium hypochlorite, Interaction, Root canal irrigation, Smear layer, Antimicrobial activity, Medicaments

## Abstract

**Introduction:**

As the root canal system considered to be complex and unpredictable, using root canal irrigants and medicaments are essential in order to enhance the disinfection of the canal. Sodium hypochlorite is the most common irrigant in endodontics. Despite its excellent antimicrobial activity and tissue solubility, sodium hypochlorite lacks some important properties such as substantivity and smear layer removing ability.

**Objective:**

The aim of this review was to address benefits and drawbacks of combining sodium hypochlorite with other root canal irrigants and medicaments.

**Discussion:**

According to the reviewed articles, NaOCl is the most common irrigation solution in endodontics. However, it has some drawbacks such as inability to remove smear layer. One of the drawbacks of NaOCl is its inability to remove the smear layer and lack of substantivity.

**Conclusion:**

The adjunctive use of other materials has been suggested to improve NaOCl efficacy. Nevertheless, further studies are required in this field.

## 1 INTRODUCTION

Due to various anatomical complexities of the canal system, mechanical preparation of the canal may reduce the bacterial load inside the canal 100-1000 folds. However, due to the fact that 35 to 50% of the surface area of the canal system remains untouched by endodontic instruments using an appropriate root canal irrigantis necessary in order to improve disinfection of the root canal system [1].

In ideal form, irrigants should have antimicrobial action, tissue-dissolution activity, demineralization, lubrication, and ability to removing smear layer and debris [2].

## 2 RATIONALE FOR COMBINING SODIUM HYPOCHLORITE (NaOCl) AND OTHER IRRIGANTS

As NaOCl has excellent antimicrobial action and also tissue solubility, it has been used in endodontics as the most common irrigation solution. NaOCl is available as 1-15% aqueous solutions with alkaline pH (around 11) [3].

One important drawback of NaOCl is its inability forsmear layer removal [3]. Therefore, adjunctive use of a chelating agent like ethylene-diamine tetra-acetic acid (EDTA) or citric acid (CA) has been proposed in order to remove the smear layer created after root canal preparation [4]. EDTA is used at10-17% concentrations; its pH is modified from original value of 4 to values up to 8 for increasing its chelating activity [5]. CA is also an organic acid normally used in endodontic therapy at 10-50% concentrations with pH 1-2 [6].

One of the other drawbacks of NaOCl is the lack of substantivity [7]. Substantive antimicrobial agents are attached to hydroxyapatite containing tissues (*i.e*. dentin) and are released gradually. Chlorhexidine (CHX) is a cationic bis-guanide with residual antibacterial activity. Combination of NaOCl and CHX has been proposed to increase their antibacterial action [8].

The antibacterial effect of NaOCl can be achieved in two different ways. A high concentration of chloride ions produces high cytotoxicity, which explains the excellent antibacterial effect. But at a lower pH, the high proportion of hypo chloric acid is explanation of the antibacterial effect. The fact that the proportion of chloride ions decreases in the solution does not mean that the solution will lose its antibacterial effect [2-4].

## 3 INTERACTION BETWEEN NaOCl AND EDTA

Adding EDTA to NaOCl can decrease the pH of NaOCl in a time-dependent manner. This may affect free chlorines in solution and increase in chlorine gas and hypochlorous acid, subsequently decreases the hypochlorite ion [9, 10].

Equal proportions of 1-2% NaOCl with 17% EDTA may result in pH of 8.0 from an initial value of 10 after 48 hours. However, when mixed in a 1:3 ratio, pH was stable during 48-hour time, possibly because of immediate interaction between solutions [11]. Baumgartner and Ibay [12] showed that pH reduction in NaOCl may cause chlorine gas release. This means that adding EDTA to NaOCl may cause lower levels of chlorine gas.

Zehnder *et al*. [10] concluded that when NaOCl was combined with EDTA, free chlorine may decrease to 0 in period of 60 minutes. Clarkson *et al*. [13] are in agreement with this conclusion. They also showed that the available chlorine loss may be 80%.

Girard *et al*. [14] evaluated interactions of gel-type preparations of chelators containing 10% urea peroxide and 15% EDTA with 1% NaOCl. Using spectroscopy, they showed that both compounds depleted the solution from chlorine after only 5 minutes. Grawehr *et al.* [15] concluded that EDTA may cause NaOCl to lose its tissue-dissolving ability. NaOCl cannot decrease calcium chelating or smear layer removal of EDTA and CA [4].

Saquy *et al*. [16] evaluated the calcium chelation ability of combination of distilled water and 17% EDTA and combination of 0.5% NaOCl and 17% EDTA and concluded that more amounts of calcium chelation can occur in solutions containing NaOCl. This study also revealed that the addition of NaOCl to EDTA cannot alter demineralization ability of EDTA. Two studies have shown that if the original free chlorine values were modest, chelators can decrease the antimicrobial activity of NaOCl [10, 15]. Grawehr *et al*. [15], by using agar diffusion test for evaluating the antimicrobial activity against Enterococcus faecalis and Candida albicans showed that NaOCl can produce smaller zones of inhibition comparing EDTA or mixture of NaOCl/EDTA.

## 4 INTERACTION BETWEEN CHX AND NaOCl

The combination of CHX and NaOCl has the ability to increase their antibacterial characteristics. Furthermore, CHX has a unique property named substantivity (prolonged antimicrobial activity) [8]. The association of CHX with NaOCl may result in a chemical smear layer which may cover dentinal tubules and so interfere with the sealing property. Moreover, this precipitate shows cytotoxicity and may change the tooth color [9].

One study using electrospray ionization concluded that 2% CHX may rapidly produce orange-brown precipitate in combination with 1% and 5.25% NaOCl, and orange-white precipitate in combination with 0.16% NaOCl [16]. It has been proposed that oxidizing activity of NaOCl causes chlorination of CHX [18]. On the other hand, some other studies, using different methodologies, failed to detect it [16-20]. Para-chloroaniline (PCA) seems to be mutagenic and cytotoxic. There are some concerns regarding the possible carcinogenicity [9].

Some studies have been done to show the chemical composition of the flocculate created by association of CHX with NaOCl [17-22]. Marchesan *et al*. [21] combined different concentrations of CHX (0.2% - 2%) and NaOCl (0.5%, 2.5%, and 5%) and showed the formation of brownish flocculate when solutions made contact with each other.

Using nuclear magnetic resonance, Krishnamurthy and Sudhakaran [23] detected PCA following mixing 2.5% NaOCl with 2% CHX. Chhabra *et al*. [24] showed that PCA was a toxic and carcinogenic substance. Using environmental scanning electron microscopy, one study assessed the influence of irrigation using on debris removal and patent dentinal tubules *ex vivo*. They showed a decrease in count of patent dentinal tubules in coronal and middle thirds of the canal between irrigation with irrigation with 5.25% NaOCl and 5.25% NaOCl/2% CHX [25]. Using scanning electron microscopy, Valera *et al*. [26] studied the percentage of closed and open tubules after canal preparation using 2.5% NaOCl and 2% CHX, intercalated by normal saline. They concluded that 2% CHX gel may produce the largest amount of open dentinal tubules. Using Rhodamine leakage, Akisuke *et al*. [27] concluded that the combination of CHX and NaOCl may cause a decrease in permeability only in the apical area. Vivacqua-Gomes *et al*. [28] also showed that a precipitate formed when combining 2% CHX gel and 1% NaOCl may enhance dye penetration in obturated canals. A recent study showed that precipitates formed when NaOCl and CHX contact did not show mutagenic and carcinogenic potential [29].

In conclusion, combination of CHX and NaOCl may cause some color changes and also the formation of an insoluble precipitate may interfere with canal seal. The canal system can be dried using sterile paper points before the final rinse by CHX.

## 5 INTERACTION BETWEEN NaOCl AND Alexidine (ALX)

ALX is a disinfectant with greater affinity for major virulence factors than CHX [30]. Kim *et al.* [31] showed that the interaction of ALX and NaOCl cannot help for the formation of PCA which is an insoluble precipitate. The color of the reacted solution changed transparent with decreasing ALX concentration. Ruiz-Linares *et al*. [32] in 2017 showed that comparing 2% ALX, 2.5% NaOCl killed bacteria significantly more efficiently when used against polymicrobial mature biofilm. Cetrimide improved the antimicrobial activity of CHX and ALX. In another study published in 2017, Bukhary and Balto [33] showed greater antimicrobial activity of NaOCl against E. faecalis compared with CHX and ALX.

## 6 INTERACTION BETWEEN NaOCl AND CA

It has been shown that good results may be obtained by the association of NaOCl to CA in permanent teeth [34-36]. This combination may allow better adaptation of root canal filling materials to the canal walls. Furthermore, this application may results in better disinfection of the canals compared to usage of each of them used separately [37]. This may be due to the fact that CA can open dentine tubules, thus results in better penetration of filling materials [36, 38, 39].

Zehnder *et al.* [40] concluded that the addition of 10% CA to 1% NaOCl may result in pH between 1.8 and 4.3. They also showed that when NaOCl was mixed with CA, free chlorine decreased immediately. Baumgartner and Ibay [41] showed that when CA was combined with NaOCl, more chlorine may be detectable and also present at further distance compared to adding EDTA.

## 7 INTERACTION BETWEEN NaOCl AND Maleic Acid (MA)

MA is used as a conditioner in dental adhesives [42]. For smear layer removal, it has been shown that 7% MA is significantly better than 17% EDTA [5]. Also, 7% MA is less cytotoxic comparing 17% EDTA [43].

There is only one study on the interaction between NaOCl and MA. Chandak *et al*. [44] showed that the combination of 7% maleic acid and 5% NaOCl significantly reduced the available free chlorine.

## 8 NaOCl AND SURFACTANTS

High surface tension is one of the major drawbacks of NaOCl. Adding surfactants increase the ability of NaOCl to penetrate the main canal and canal irregularities *in vitro* [45, 46]. Combination of NaOCl and ethanol moved further in the capillary tubes comparing NaOCl alone, depending on the amount of ethanol in the combinations [46].

Stojicic *et al*. [47] concluded that NaOCl combined with surface active agents shows lowest contact angle on dentin and is most effective in dissolution of the soft tissue. Another study demonstrated that the addition of surfactant alone did not appear to improve the abilities of NaOCl to dissolve dental pulp tissue [48]. Williamson *et al*. [49] revealed that adding 0.1% cetrimide to 2% NaOCl increased its antibacterial activity.

Another issue is the effect of surfactants on the stability of NaOCl. Adding surfactants modifies the stability of NaOCl [46, 50]. Ethanol reduces the free available chlorine (FAC). Adding 50% ethanol to 2% NaOCl, the solutions are almost depleted from their FAC in 15 min; however, 30% ethanol combination shows 70% loss after 30 min [46]. Clarkson *et al*. [51] evaluated influence of surfactants on chlorine loss of NaOCl due to interactions with EDTA and concluded that products containing surface-active agents show lower FAC reduction at some dilutions. Studies regarding the effect of surfactants on the tissue solubility of NaOCl are controversial.

Cameron *et al*. [50] showed that surfactant had no significant effect on the tissue dissolving ability of NaOCl. Furthermore, another study showed that reduced surface tension cannot cause greater soft tissue dissolution by NaOCl [52]. However, a study revealed that NaOCl preparations containing surface active agents dissolved soft tissue more rapidly than NaOCl containing no surfactant [53]. In addition, Clarkson *et al*. [48] showed that reduced surface tension improved solubility of bovine pulp tissue.

## 9 OCTENIDINE-BASED SOLUTION

Octenisept (OCT; Schülke & Mayr, Nordersdedt, Germany) is an antimicrobial/antibiofilm agent can be potentially combined with NaOCl during root canal treatment. A recent study showed that the whitish precipitate formed with the NaOCl-OCT mixture was identified as phenoxyethanol, a compound already present in OCT, and it may occlude dentinal tubules [54].

Bukhary and Balto [33] showed greater antimicrobial activity of NaOCl against E. faecalis compared with OCT. OCT was more effective than CHX.

## 10 CHLOR-XTRA

Chlor-XTRA is a new NaOCl-based irrigation solution composed of 5.85% NaOCl and a detergent to reduce surface tension. Its appearance is clear light yellow green. It is completely soluble in water with a chlorine-like odour. It is 2.6 times more digestive than regular NaOCl. Furthermore, its wetting ability is 2.5 times greater than regular NaOCl [55]. Recently, using agar diffusion method, Mohammadi *et al*. [56] demonstrated that Chlor-XTRA was more effective against Actinomyces israelii compared to NaOCl, CHX, Tetraclean and Hypoclean.

Jungbluth *et al*. [52] showed that chemical assessment of different bottles of Chlor-Xtra has different chlorine content. Stojicic *et al.* [57] showed that Chlor-XTRA dissolved significantly more tissue than other solutions in every concentration.

## 11 HYPOCLEAN

A new modified NaOCl solution (Hypoclean) has been introduced by Giardino. This solution is a detergents-based irrigant composed of 5.25% NaOCl and 2 detergents [58]. Recently, Mohammadi *et al*. [56] showed that Hypoclean is more effective against C. albicans, P. aeroginosa, and L. casei than NaOCl, CHX, Tetraclean, and Chlor-XTRA.

## 12 SYNERGISM BETWEEN Calcium Hydroxide (Ca(OH)_2_) AND NaOCl

Root canals cannot be cleaned by physical instruments alone. Chemicals can help to supplement this procedure. Irrigation with NaOCl and intracanal placement of Ca(OH)_2_ are samples of such usage that utilize chemicals with the aim of facilitating the tissue remnant removal [59].

Some controversies have been reported regarding the synergistic effects of Ca(OH)_2_ and NaOCl. Hasselgren *et al*. [60] showed that Ca(OH)_2_ paste has the ability of tissue dissolving after 12 days. They also showed an increase in tissue dissolving of NaOCl after pretreatment with Ca(OH)_2_ for 30 minutes up to 7 days. In another study, Metzler and Montgomery [61] concluded that pretreatment with a hard-setting Ca(OH)_2_ paste for 7 days followed by irrigation with NaOCl can clean canal isthmuses better than hand mechanical preparation alone. Yang *et al.* [59] showed that both NaOCl and Ca(OH)_2_ partially dissolved pulp tissue. The anaerobic environment could not alter the tissue dissolving property. Wadachi *et al*. [62] also showed that the amount of debris was reduced in teeth treated with Ca(OH)_2_ for seven days or NaOCl for >30 seconds. Combination of NaOCl and Ca(OH)_2_ was more effective than separate protocols. On the other hand, some researches have shown that Ca(OH)_2_ may be ineffective for pulpal tissue dissolving. Morgan *et al*. [63] also showed the minor effect of Ca(OH)_2_ for tissue dissolving. In summary, it seems that pretreatment with Ca(OH)_2_ medicament may increase the tissue dissolving effect of NaOCl.

## 13 INTERACTION BETWEEN NaOCl AND MTA

MTA has been produced as gray and white MTA; both with the base of Portland cement. Hydrophilic powder needs some moisture for optimal setting. Traditionally, MTA powder is mixed with supplied sterile water in a 3:1 powder/liquid ratio. Different liquids have been suggested for mixing with the MTA powder [64-66].

Using differential scanning calorimetry, Zapf *et al*. [67] concluded that NaOCl and lidocaine may be detrimental to MTA reaction product formation. According to Camilleri [68], immersion of white MTA and bismuth oxide in NaOCl may result in dark discoloration.

Ballester-Palacios *et al*. [69] showed that 5% NaOCl significantly reduces the surface roughness of Portland cement. It also lowered the MTA roughness.

Al-Anezi *et a*l [70]. showed that adding NaOCl to MTA improved the handling properties and decreased setting time. Jafarnia *et al*. [71] assessed the effect of NaOCl on the cytotoxicity of MTA and found that 3% NaOCl increased the toxicity of MTA against L929 cell.

## CONCLUSION

According to this study and other documents [72-77], it can be concluded that:

1. Due to the excellent antimicrobial activity and tissue solubility, NaOCl is the most common irrigation solution in endodontics. However, it has some drawbacks such as inability to remove smear layer. One of the drawbacks of NaOCl is its inability to remove the smear layer and lack of substantivity. Therefore, the adjunctive use of other materials has been suggested to improve its efficacy.
2. Combining NaOCl with EDTA decreases free available chlorine dramatically. However, using EDTA or CA was suggested removing the smear layer associated with mechanical instrumentation.
3. Combination of CHX and NaOCl has been suggested to enhance their antibacterial activity and induce substantivity.
4. Combining NaOCl with MA and CA reduces free available chlorine.
5. Regarding adding surfactants to NaOCl, studies regarding the effect of surfactants on the tissue solubility of NaOCl are controversial. However, surfactants reduce free available chlorine in NaOCl solutions.
6. Pretreatment with Ca(OH)_2_ may enhance the ability of tissue dissolving of NaOCl.
7. NaOCl is detrimental to MTA reaction product formation. Furthermore, immersion of white MTA in NaOCl may result in the formation of brown discoloration. In addition, NaOCl significantly lowers the surface roughness of MTA, decreases its setting time, improves its handling properties and increases its cytotoxicity.
8. Combining NaOCl with some other irrigating solutions such as MTAD, oxygen peroxide, and strong acids (like phosphoric acid and nitric acid) should be studied in the future.
